# Pan-continental spillover risk: integrated spatiotemporal, transmissibility and surveillance analysis of avian influenza a(H5N1) in Africa

**DOI:** 10.3389/fepid.2026.1813211

**Published:** 2026-07-01

**Authors:** Maladho Diaby, Alhassane Diallo, Castro G. Hounmenou, Salifou Talassone Bangoura, Emile Faya Bongono, Kadio Jean-Jacques Olivier Kadio, Aly Badara Touré, Abdourahamane Barry, Saidouba Chérif Camara, Haby Diallo, Thibaut Armel Chérif Gnimadi, Sidikiba Sidibé, Alexendre Delamou, Alioune Camara, Alpha-Kabinet Kéita, Abdoulaye Touré

**Affiliations:** 1 Centre de Recherche et de Formation en Infectiologie de Guinée (CERFIG), Gamal Abdel Nasser University, Conakry, Republic of Guinea; 2 Department of Pharmaceutical and Biological Sciences, Faculty of Sciences and Health Techniques, Gamal Abdel Nasser University, Conakry, Republic of Guinea; 3 Department of Public Health, Faculty of Sciences and Health Techniques, Gamal Abdel Nasser University, Conakry, Republic of Guinea; 4 African Centre of Excellence in the Prevention and Control of Communicable Diseases (CEA-PCMT), Faculty of Sciences and Health Techniques, Gamal Abdel Nasser University, Conakry, Republic of Guinea; 5 TransVIHMI, IRD/INSERM/Monpellier University, Montpellier, France

**Keywords:** Africa, avian influenza, bayesian modelling, H5N1, one health, public health surveillance

## Abstract

**Background:**

The HPAI H5N1 panzootic represents a critical threat to human health in Africa, where traditional poultry systems and dense human-animal interfaces facilitate frequent zoonotic spillover. While sporadic human cases raise pandemic concerns, continent-wide integration of spatial dynamics, transmissibility indicators, and surveillance performance has been lacking. This study quantifies avian influenza transmission over two decades across Africa, identifies geographical hotspots, and evaluates the responsiveness of current surveillance systems.

**Methods:**

We analysed 8,037 avian influenza outbreak events and 369 laboratory-confirmed human cases, predominantly caused by HPAI H5N1 (2004–2025), using harmonised data from FAO (EMPRES-i+), WHO, and WOAH. A Bayesian Besag-York-Mollié (BYM) spatiotemporal model estimated residual transmission risks and Incidence Rate Ratios (IRR) by subtype. The basic reproduction number (R₀) was derived via an exponential growth model applied to human outbreak phases across infectious durations of 7–30 days. Surveillance responsiveness was assessed by quantifying notification delays between clinical observation and official reporting.

**Results:**

Risk of infection in animals: HPAI H5N1 was the dominant strain, representing 87.8% of animal cases, with Egypt acting as the primary epidemiological epicentre (66% of total records). The spatiotemporal model revealed that H5N1 is associated with a significantly higher risk of animal infection (IRR = 8.37; 95% CI: 6.65–10.53). Although 71% of outbreaks were reported within 5 days of detection, significant delays (≥15 days) occurred in 12% of cases, with notable regional disparities. Risk of infection in human: H5N1 was associated with a 67-fold increase in the incidence of human cases compared to other subtypes (IRR = 66.78; 95% CI: 25.29–176.37). Sensitivity analyses yielded R_0_ estimates ranging from 1.05 (95% CI: 0.91–1.31) to 1.23 (95% CI: 0.60–2.33), indicating localised epidemic potential.

**Conclusion:**

Our findings highlight a persistent and geographically heterogeneous H5N1 reservoir in Africa with high zoonotic affinity. Although sustained human-to-human transmission remains limited, the identification of dual poultry-human hotspots and localised R0 peaks underscores the urgent need for geographically targeted One Health interventions. Strengthening real-time reporting systems and improving biosecurity in high-risk poultry value chains are critical to mitigating future pandemic threats on the continent.

## 1 Introduction

Avian influenza, particularly the high pathogenicity H5N1 strain, poses a significant threat to human, animal and environmental health worldwide. Now panzootic in poultry, the virus continues to spread globally, increasing the risk of zoonotic transmission, although human-to-human transmission remains limited (1, 2). The current panzootic is driven by the clade 2.3.4.4b, which has provoked unprecedented mortality in both wild and domestic birds across numerous countries since 2020, with over 1,400 outbreaks reported in 42 countries, confirming the persistence and global reach of viral circulation (3, 4). The high viral replication capacity and adaptive potential of the virus raise serious concern, particularly in Africa, where vulnerability to avian influenza spread is exacerbated by traditional agricultural systems, inadequate biosecurity practices, informal transportation networks, and frequent close interactions among humans, domestic poultry, and wildlife, often occurring in the context of increasing encroachment into natural ecosystems (5–9). These factors alter the viral ecology and facilitate transmission between domestic poultry, wild birds and humans (10). Wild birds serve as a significant reservoir and vector, threatening the stability of African poultry farms.

A defining and increasingly alarming feature of the current panzootic is the rapid genetic evolution of clade 2.3.4.4b through extensive reassortment events. Recent genomic sequencing has revealed the emergence of multiple novel genotypes, with intercontinental dissemination primarily mediated by migratory wild birds (11, 12). These reassortment dynamics have enabled the virus to breach previously stable species barriers: most notably, HPAI H5N1 clade 2.3.4.4b has been confirmed in dairy cattle in the United States, representing a paradigm shift in our understanding of influenza A host plasticity (13). Cross-species transmission has been further evidenced by catastrophic mortality events in marine mammals, with mass die-offs in South American elephant seal colonies providing direct epidemiological evidence of sustained mammal-to-mammal transmission, driven by PB2 adaptive mutations identified as key molecular determinants of this expanded host tropism (14, 15).

Recurring outbreaks of avian influenza have severely affected the poultry sector and food security in many countries. Ghana, Nigeria, and Ethiopia have suffered substantial poultry losses, with economic impacts estimated in the millions of US dollars (16–20). In 2022, Guinea's poultry sector was affected by an outbreak of high pathogenicity H5N1 avian influenza, which recorded an average mortality rate exceeding 40% of its poultry population (21). These epidemics compromise food security, exacerbate poverty, and undermine the livelihoods of small-scale producers (22, 23). Poultry production, a pillar of nutritional security in many rural communities, remains highly exposed to zoonotic risks.

In light of this threat, strengthening biosecurity measures is crucial. Standardised hygiene protocols, waste management, poultry movement control, and quarantine measures are essential during outbreaks (24, 25). Training and awareness campaigns for poultry farmers are equally important (21, 26–29). Effective surveillance systems, rapid diagnosis well-equipped laboratories, qualified personnel, real-time data collection and cross-sector coordination between human and animal health services are needed for early outbreak detection and prevention of human transmission (26, 30).

According to data published in 2026 by the World Health Organisation (WHO), 1,000 laboratory-confirmed human cases of H5N1 virus infection, resulting in 479 deaths, have been reported in 25 countries worldwide since 2003. In Africa alone, 361 cases of infection have been reported, including 121 deaths, highlighting a high mortality rate on that continent (31). This has led to the inclusion of H5 influenza viruses on the WHO's priority list for epidemic preparedness research (32). Most human infections result from direct contact with infected poultry or their products, particularly during handling, preparation or consumption. Live poultry markets are high-risk exposure settings (2, 33).

Although numerous studies have described the epidemiology of avian influenza in animal and human populations, integrated analyses combining spatio-temporal dynamics, surveillance system performance and transmissibility indicators remain limited in Africa. This study aimed primarily to estimate the risk of avian influenza virus transmission to humans in Africa by combining spatio-temporal analysis, estimation of the basic reproduction number (R0), and an examination of notification delays.

## 2 Materials and method

### 2.1 Data sources

The data were harmonized and extracted from the Animal Health Emergency Prevention System (EMPRES-i+), the epidemiological surveillance databases of the Food and Agriculture Organisation of the United Nations (FAO), as well as from the World Organization for Animal Health (WOAH), the, and the World Health Organisation (WHO). The dataset consisted of a comprehensive longitudinal record (2004–2025) including the year, sub-region, country, administrative area, and locality, along with geolocation, subtype, source of diagnosis and status, animal type and species, notification date, reporting date, number of human cases and deaths. The integration of these multiple institutional sources was performed to minimise reporting bias and ensure continent-wide coverage. The analyses included confirmed animal and human cases documented between 2004 and 2025. The final data extraction was performed on 22 March 2025.

### 2.2 Data analysis

Categorical variables were summarised as frequencies and percentages, and quantitative variables were expressed as medians with interquartile ranges. Reported avian influenza cases were described by subtype, geographical region, host species, and temporal distribution. Case counts and proportions were computed for animal and human infections separately to characterise the overall epidemiological burden across the African continent over the study period (2004–2025).

To account for spatial autocorrelation and potential under-reporting, the adjusted analysis employed a Bayesian hierarchical Besag-York-Mollié (BYM) model (34). This model estimates the residual spatial risk that cannot be explained by geographical proximity alone, effectively smoothing estimates across adjacent administrative units.

We estimated expected cases per country/year using Bernardinelli's model (35). This model assumes that the number of observed cases $Y_{i,t,s}$ (in country *i*, year *t*, for subtype *s*) follows a Poisson distribution: $Y_{i,t,s}∼\mathrm{Poisson}({\lambda \;}_{i,t,s})$ where ${\lambda \;}_{i,t,s}$ is the expected mean, defined as: $\mathrm{Log}\;(\lambda i,t,s)=\;\mu +u_{i}+v_{i}+\delta_{s}+\eta_{i,t}$.

Where *μ* is the global intercept; $u_{i}$ represents the country-specific random effect (structured spatial effects); $v_{i}$ denotes the global linear time trend (unstructured spatial effects); *δ_s i_* indicates subtype fixed effects, and $\eta_{i,t}$ is the space-time interaction term (difference between global and country-specific trends).

The magnitude of association between each subtype and infection risk was expressed as an Incidence Rate Ratio (IRR), defined as the ratio of the incidence rate of a given subtype to that of the reference subtype (H5 HPAI): IRR = *λ*₁/λ₀, where *λ*₁ is the incidence rate of the subtype under study and *λ*₀ is the incidence rate of the reference subtype. An IRR greater than 1 indicates an increased incidence rate relative to the reference subtype, an IRR less than 1 indicates a reduced rate, and an IRR equal to 1 indicates no differential risk. Posterior estimates were summarised as posterior means with 95% credible intervals (95% CrI). A subtype effect was considered statistically significant when the 95% CrI excluded the null value of 1. For interpretability, the relative percentage difference in incidence compared to the reference was calculated as (1 − IRR) × 100 for IRR < 1, and (IRR − 1) × 100 for IRR > 1.

We used the exponential growth method (36) to estimate the basic reproduction number ($R_{0}$) for human outbreaks (37, 38). The growth rate r was calculated based on the initial epidemic phase to capture early-stage transmissibility potential. The $R_{0}$ was derived using a simple Susceptible-Infected-Recovered (SIR) model: $R_{0}=1+r*D$, where *D* is the duration of infection. In sensitivity analysis, we varied the infection duration (7, 14, 21, and 30 days) to assess the stability of the results against biological uncertainty.

Concerning the notification delays analysis, the time between case observation and reporting was calculated to assess surveillance responsiveness. All analyses were performed using an R package (version 4.4.0), utilizing the INLA package for Bayesian inference.

### 2.3 Ethical considerations

This study used aggregated, anonymized data from public surveillance systems and did not require ethical approval.

## 3 Results

### 3.1 Animal outbreak data analysis

#### 3.1.1 Overall description of reported outbreak events in the animal population

A total of 8,037 avian influenza outbreak events were recorded in the animal population on the African continent between 2004 and 2025. The highly pathogenic H5N1 subtype (HPAI) demonstrated overwhelming dominance, accounting for 7,042 outbreak events and representing 87.8% of all animal reports. Other circulating lineages included H5N8 HPAI (372 outbreak events; 4.6%), H5N2 LPAI (86 outbreak events; 1.1%), H5N2 HPAI (83 outbreak events; 1.0%), and H9N2 LPAI (112 outbreak events; 1.4%), with other strains present in negligible proportions (Table 1).

**Table 1:** Description of reported cases of avian influenza in Africa, 2004–2025.

**Characteristics**	***n* (%)**
Subregion (*N* = 8037)	
Eastern Africa	28 (0.3)
Middle Africa	68 (0.8)
Northern Africa	5,586 (69.5)
Southern Africa	965 (12.0)
Western Africa	1,390 (17.3)
Subtype	
HPAI H5N1	7,042 (87.8)
H5N8 HPAI	372 (4.6)
H9N2 LPA	112 (1.4)
H7N6 HPAI	90 (1.1)
H5N2 LPAI	86 (1.1)
H5N2 HPAI	83 (1.0)
H5 HPAI	76 (0.9)
H7 LPAI	34 (0.4)
H7 HPAI	29 (0.4)
H7N1 HPAI	24 (0.3)
Other	69 (0.8)
Unknow	14 (0.2)
Animal type (*N* = 7,670)	
Captive	14 (0.2)
Domestic	7,324 (95.5)
Environmental sample	8 (0.1)
Wild	324 (4.2)
Species (*N* = 7,856)	
Other	506 (6.6)
Chicken	2,790 (36.4)
Duck	1,286 (16.8)
Goose	187 (2.4)
Ostrich	291 (3.8)
Turkey	164 (2.1)
Unspecified Bird	2,632 (34.3)
Diagnosis status	
Confirmed	8,005 (96.0)
Denied	32 (0.4)

Geographically, North Africa emerged as the primary epidemiological epicenter, accounting for 5,586 outbreak events (69.5%), followed by West Africa (1,390 outbreak events; 17.3%), Southern Africa (965 outbreak events; 12.0%), Central Africa (68 outbreak events; 0.8%), and East Africa (28 outbreak events; 0.3%). Egypt reported the highest burden, with 5,308 outbreak events representing 66.0% of all events recorded continent-wide, followed by Nigeria (999 outbreak events; 12.4%) and South Africa (951 outbreak events; 11.8%) (Table 1).

Regarding host distribution, the majority of outbreak events (7,324; 95.5%) occurred in domestic poultry, while wild birds accounted for 4.2% (324 outbreak events). Chickens were the most affected taxa (2,790 outbreak events; 36.4%), followed by ducks (1,286 outbreak events; 16.8%). Notably, a substantial proportion of records (2,632 outbreak events; 34.3%) lacked specific taxonomic identification, highlighting a significant gap in field-level species reporting (Table 1).

#### 3.1.2 Spatiotemporal analysis and risk of infection in animals

**Figure 1:**
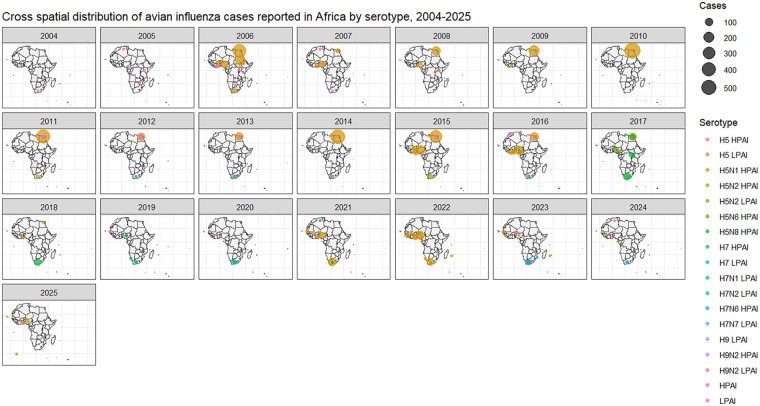
Gross spatial distribution of avian influenza cases reported in Africa by subtype, 2004–2025.

The spatial distribution of avian influenza cases reported in Africa by subtype highlighted notable differences between countries, with certain subtypes appearing to predominate in specific geographical areas (Figure 1).

**Figure 2:**
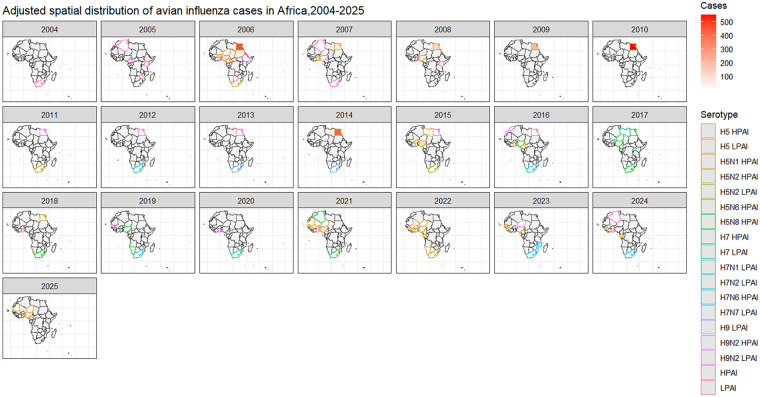
Adjusted spatial distribution of avian influenza cases in Africa, 2004–2025.

After adjusting for spatial proximity and data structure, certain regions maintained a high probability of animal cases, distinguishing genuine epidemiological hotspots from areas with high notification rates (Figure 2).

The Poisson spatio-temporal model revealed significant variations in incidence rates by subtype. HPAI H5N1 was associated with an eight-fold increase in the risk of animal infection (IRR = 8.37; 95% CI: 6.65–10.53) compared to the H5 HPAI reference, followed by H7N6 HPAI (IRR = 2.092, 95% CI: 1.543–2.836. Conversely, H5N2 HPAI (IRR = 0.209) and H9N2 LPAI (IRR = 0.281) presented significantly lower risks (Table 2).

**Table 2:** Estimation of animal infection risk in Africa according to subtype, 2004–2025.

Reference category: H5 HPAI				
**Subtype**	**IRR**	**95% CI (Lower)**	**95% CI (Upper)**	***p*-value**
H5 LPAI	0,017	0,006	0,046	0,000
HPAI H5N1	8,37	6,645	10,533	0,000
H5N2 HPAI	0,209	0,154	0,283	0,000
H5N2 LPAI	1,281	0,94	1,746	0,115
H5N6 HPAI	0,042	0,006	0,314	0,002
H5N8 HPAI	0,851	0,664	1,09	0,202
H7 HPAI	0,739	0,482	1,133	0,165
H7 LPAI	0,591	0,387	0,903	0,015
H7N1 HPAI	0,631	0,399	0,999	0,049
H7N1 LPAI	0,366	0,210	0,637	0,001
H7N2 LPAI	0,152	0,046	0,499	0,001
H7N6 HPAI	2,092	1,543	2,836	0,000
H7N7 LPAI	0,269	0,149	0,484	0,000
H9 LPAI	0,028	0,013	0,061	0,000
H9N2 HPAI	1,924	0,26	14,262	0,522
H9N2 LPAI	0,281	0,208	0,380	0,000
HPAI	0,982	0,572	1,686	0,949
LPAI	0,253	0,018	3,53	0,307

The temporal analysis also shows a decrease in risk over time, while the spatial effect highlights significant disparities between countries (Table 3).

**Table 3:** Spatio-temporal variability of animal infection in Africa, 2004–2025.

Effect	Precision	Variance	Standard deviation
Spatial	0.01383	72.34	8.50
Temporal	0.29063	3.44	1.86

#### 3.1.3 Surveillance performance and transmibility

**Figure 3:**
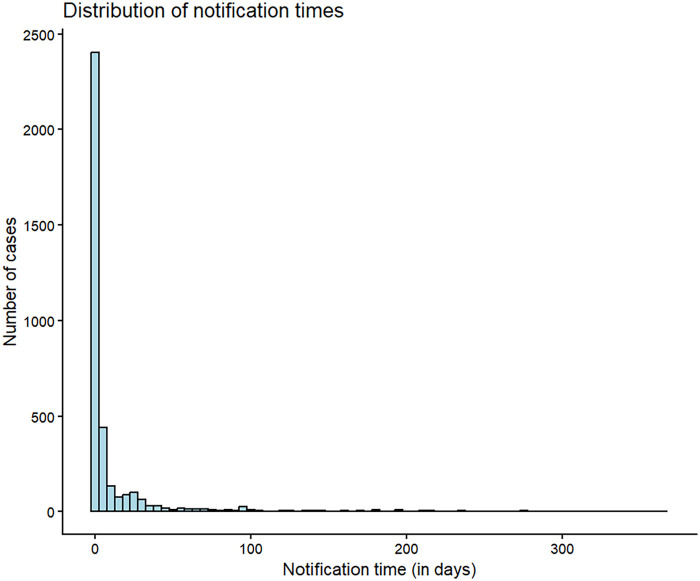
Distribution of notification times according to the number of observed cases of animal infection in Africa between 2004 and 2025.

The majority of animal cases were reported within 0–5 days of clinical observation. However, the distribution of reporting delays was highly asymmetrical, with a “long tail” showing delays of up to several weeks in specific regions (Figure 3).

### 3.2 Human outbreak data analysis

#### 3.2.1 Description of human infection cases and deaths

**Figure 4:**
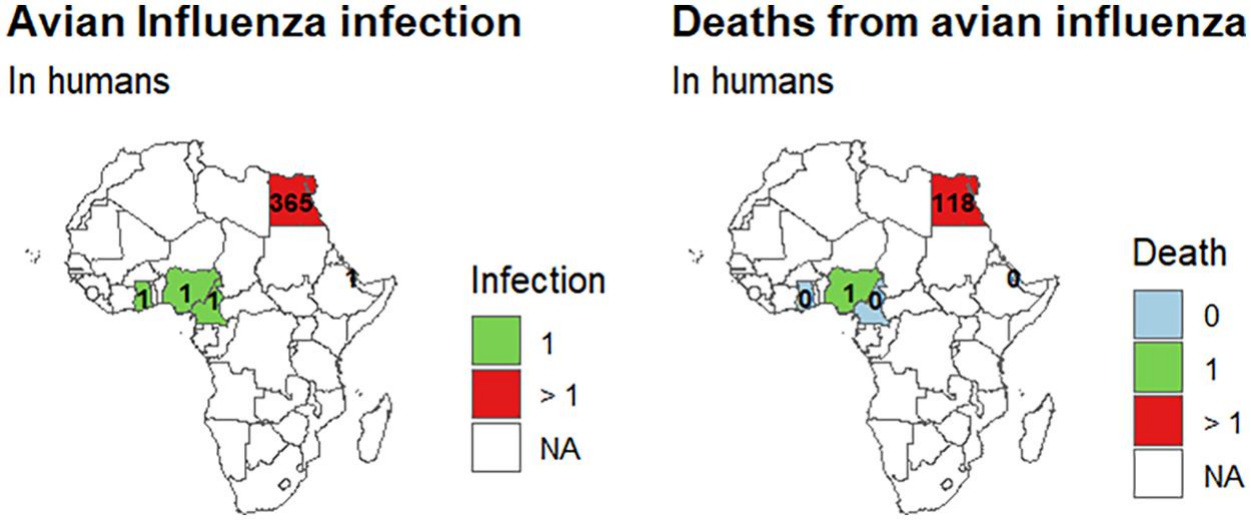
Description of human cases of infection and deaths from avian influenza in Africa, 2004–2025.

A total of 369 laboratory-confirmed human infections were documented, with the vast majority (364 cases, 98.6%) caused by HPAI H5N1. These cases were primarily linked to direct contact with infected poultry. While some familial clusters were identified, these have been investigated as point-source infections resulting from shared exposure to contaminated poultry environments rather than evidence of sustained human-to-human transmission. Human spillover events were heavily clustered in North Africa, particularly in Egypt, which accounted for 365 cases (98.9%). Other countries (Cameroon, Djibouti,Ghana and Nigeria) reported sporadic single-case events linked to H5N1. Deaths showed a similar concentration in Egypt (99.2 of the 119 deaths), reflecting a case fatality rate (CFR) of approximately 32.2% across the continent (Figure 4).

#### 3.2.2 Spatiotemporal analysis and risk of infection in human

**Figure 5:**
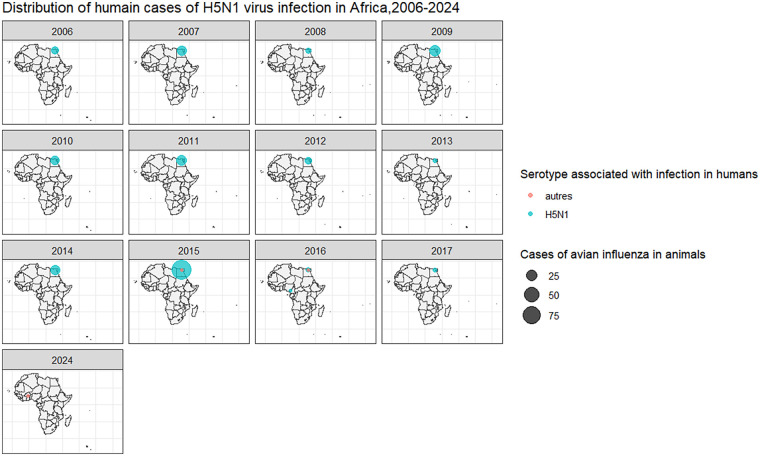
Distribution of human cases of H5N1 virus infection in Africa, 2006-2024.

The Bayesian spatiotemporal model identified clear geographical dual hotspots, where high-risk areas for poultry and humans significantly co-located (Figure 5).

For human infection, the model showed a stark and significant association between subtype H5N1 and the incidence rate. The risk of human infection with H5N1 was approximately 67 times higher than that of other circulating subtypes (IRR = 66.78; 95% CI: 25.29–176.37) (Table 4). Hyperparameter analysis showed significant spatial variability across countries (variance = 10.75) compared with temporal variability (variance = 6.61), suggesting that local environmental and systemic factors are the dominant drivers of zoonotic spillover (Table 5).

**Table 4:** Estimation of the risk of human infection with the H5N1 virus in Africa between 2004 and 2025.

Subtype	IRR	IC95_low	IC95_high
Intercept	0.33	0.03	3.21
H5N1	66.78	25.29	176.37

**Table 5:** Spatio-temporal variability of human infection with the H5N1 virus in Africa between 2004 and 2025.

Parameter	Precision	Variance	Standard deviation
Spatial	0.093	10.75	3.28
Temporel	0.151	6.61	2.57

#### 3.2.3 Basic reproduction number in humans

Transmissibility estimates ($R_{0}$) for human outbreaks increased progressively with longer assumed infection durations. For a 7-day duration, ($R_{0}$) was 1.05 (95% CI: 0.91–1.31), reaching a maximum of 1.23 (95% CI: 0.60–2.33) for a 30-day duration. The consistency of $R_{0}$ values above 1.0 in specific cluster phases indicate transient episodes of amplified transmission potential, despite the absence of sustained pandemic spread (Table 6).

**Table 6:** Estimated reproduction rate according to different infection durations.

**Time (day)**	**R0**	**IC95%**
7	1.05	[0.91–1.31]
14	1.11	[0.81–1.63]
21	1.16	[0.72–1.93]
30	1.23	[0.60–2.33]

## 4 Discussion

This study provides a comprehensive quantification of the risks of avian influenza virus transmission to humans in Africa over a critical 20-year period. Data from African countries showed a substantial cumulative burden in both birds and humans between 2004 and 2025. This is consistent with observations from Szablewski et al. (39) which describes a global trend towards an increase in the detection of avian influenza in animals and associated human cases, the latter generally remaining low in number but severe. However, our findings reveal stark geographical heterogeneity and a diversity of subtypes, suggesting that regional drivers of spillover are more complex than previously understood.

It is important to note that the zoonotic landscape has shifted significantly since 2016. The high number of human infections observed between 2006 and 2014 was largely driven by the endemic circulation of the H5N1 lineage 2.2.1 in Egypt. The subsequent emergence and continent-wide expansion of clade 2.3.4.4b, while responsible for unprecedented poultry mortality, has shown a distinct epidemiological profile in humans compared to the earlier period. Our temporal analysis captures these aggregated fluctuations, though future studies focusing specifically on pre- and post-2016 genomic data are required to fully disentangle these distinct evolutionary drivers.

This heterogeneity is particularly evident in North Africa, which emerged as the continent's primary epicenter, reflecting not only high poultry density and the hyper-endemic H5N1 situation in Egypt, but also its position at the convergence of major migratory flyways connecting Eurasian breeding grounds with African wintering areas. Phylogenetic analyses suggest that these flyways act as both sources and sinks for avian influenza viruses, enabling intercontinental dissemination, with recent evidence documenting a progressive east-to-west spread of clade 2.3.4.4b across North and West Africa. East Africa, despite its low reported burden, sits along the East African-West Asian flyway, where southward autumn migration of waterfowl plays a critical role in cross-continental H5N1 transmission, suggesting that its low outbreak counts likely reflect surveillance gaps rather than genuine absence of viral circulation (40, 41).

HPAI H5N1 demonstrates significant capacity for transmission and persistence within avian populations across the continent. This sustained circulation in animals constitutes a key driver of zoonotic risk. At the animal level, our spatiotemporal model quantifies the relative dominance of H5N1 with an IRR of 8.37, indicating a substantially higher risk of animal infection compared to other co-circulating strains like H9N2 or H5N8. This suggests that while other strains may pose significant economic threats to the poultry sector, H5N1 remains the primary bridge for human infection in Africa (42, 43). While this finding is consistent with the established literature, its value lies in providing a quantified, continent-scale confirmatory estimate anchored to 21 years of surveillance data, a baseline against which future shifts in zoonotic risk can be measured (44, 45). Indeed, what matters epidemiologically is not the point estimate alone, but its trajectory over time (44): a rising IRR for H5N1 across successive time windows would signal increasing bridging efficiency between animal reservoirs and human hosts, potentially reflecting viral adaptation, ecological shifts, or widening surveillance gaps (45).

In the human population, the strong correlation observed between H5N1 and the frequency of infection cases highlights its increased zoonotic potential. The high spatial variability, as opposed to temporal variability, indicates that the risk to humans is mainly influenced by geographical and contextual determinants, such as informal value chains and Live Poultry Markets (LPMs) (46, 47). This implies that pandemic preparedness in Africa must shift from a “reactive seasonal” approach to a “geographically-targeted risk” model, focusing on these persistent dual hotspots (48).

The gradual increase in R₀ estimates as the duration of infection lengthens is consistent with the principles of infectious disease dynamics (49, 50). Notably, R₀ varies substantially across host species, with transmission dynamics in wild birds where H5N1 circulates largely asymptomatically differing markedly from those in domestic poultry, where high host density and biosecurity gaps drive higher effective R₀ values (40, 41). The values estimated in this study (R₀ = 1.05 to 1.23) are consistent with previous studies describing the H5N1 virus as having borderline epidemic potential (2, 51, 52), suggesting a low potential for sustained spread in the general population. Human infections thus remain largely sporadic and dominated by zoonotic events, as also reported by Mohamed et al. (53) with estimates of R₀ below 0.2 in comparable contexts. Crucially, while our median estimates align with a lack of sustained pandemic spread, the upper bounds of our R₀ sensitivity analysis (up to 2.33) during specific outbreak phases suggest that localized clusters of human-to-human transmission remain a biological possibility (54). These transient episodes of amplified risk require heightened vigilance, particularly in family clusters or settings with intense exposure (54). The recent expansion of clade 2.3.4.4b into dairy cattle herds across the United States, the first documented sustained mammal-to-mammal transmission of this clade in a terrestrial livestock species, demonstrates that host range boundaries previously considered stable can be breached rapidly through the acquisition of PB2 mammalian adaptation mutations (13, 55), and serves as a reminder that R₀ trajectories are not static across the evolutionary arc of the virus. In general, meta-analyses confirm that the R₀ of H5N1 remains below 1 on average in most epidemiological contexts (56, 57), thus limiting its pandemic potential but highlighting the need for increased vigilance and enhanced surveillance, particularly during phases of rapid epidemic growth (2, 58, 59).

Notification times showed an asymmetrical distribution. While the median delay of 5 days is relatively responsive, the presence of significant regional lags is a major concern. The literature confirms that these delays can compromise the effectiveness of interventions. In a study conducted in 2018, Hill and al. showed that active surveillance systems on poultry farms can reduce the notification time from 7 to 2 days, thereby significantly improving the response (60).

Furthermore, a study on the performance of reporting outbreaks of avian influenza at the global level highlights significant inter-country and inter-sectoral variability in the time taken to report HPAI alerts (61). In the context of the current 2021–2025 panzootic wave, such delays may allow for the “silent spread” of the virus in poultry populations, increasing the window for potential human exposure and viral reassortment before control measures are implemented.

Our results call for a fundamental reinforcement of African avian influenza strategies. Efforts should move beyond simple culling policies toward an integrated One Health approach that brings together epidemiological surveillance, risk communication, and community engagement. Operationalizing One Health in this context requires real-time data integration between veterinary, public health, and wildlife services to bridge the reporting gaps identified in our spatiotemporal model. Wildlife professionals including ecologists, conservation biologists, and wildlife veterinarians must be structurally integrated into national and regional avian influenza response frameworks, given the demonstrated role of wild birds as intercontinental vectors of clade 2.3.4.4b and the entirely unmonitored exposure risk posed by free-ranging mammals, including non-human primates, in forest-edge and peri-urban ecosystems across the continent.

## 5 Strengths and limitations

The principal strength of this study lies in its comprehensive, longitudinal scope covering two decades (2004–2025) across the entire African continent. By integrating spatiotemporal modeling with transmissibility metrics (R_0_) and surveillance performance data, this analysis provides a multifaceted view of the zoonotic landscape that was previously unavailable. Despite this, several limitations must be acknowledged.

First, the absence of precise information on the duration of infection in human cases introduces uncertainty into the R_0_ calculations. However, we addressed this by conducting a rigorous sensitivity analysis across a biological spectrum of 7–30 days, ensuring that our transmissibility estimates remain robust across different clinical scenarios.

Second, significant heterogeneity within the exposed populations and reporting biases across countries could not be fully eliminated. Nevertheless, the application of a rigorous Bayesian Besag-York-Mollié (BYM) model effectively mitigates these inconsistencies by smoothing risk estimates across adjacent geographical units and accounting for structured spatial random effects. This approach has been previously validated in infectious disease surveillance contexts subject to variable reporting completeness; for instance, Guo et al. extended the BYM model to spatiotemporal influenza surveillance data from a national notifiable disease reporting system, demonstrating its robustness in settings with heterogeneous case ascertainment across regions (62).

Furthermore, the surveillance data are heavily influenced by the intensive monitoring systems in Egypt, which may mask underlying transmission dynamics in countries with less robust reporting. While our Bayesian Besag-York-Mollié (BYM) model effectively accounts for spatial autocorrelation and structural noise, the disparity in surveillance density remains a significant challenge, necessitating cautious interpretation of regional risk estimates outside of North Africa.

Third, the potential influence of seasonality on viral circulation was not explicitly quantified in this multi-decadal analysis. In addition, the analysed data are inherently subject to the limitations of passive surveillance systems. While under-reporting is a known challenge in global health, our use of harmonized institutional data from FAO-EMPRES, WHO, and WOAH represents the most validated evidence currently available for the region. The utilization of Bayesian inference further strengthens the inferential power of our findings by incorporating uncertainty into the risk maps.

## 6 Conclusion

Combined analysis of spatio-temporal variations, notification delays basic reproduction number (R₀) highlights the unprecedented complexity and heterogeneity of avian influenza ecology in Africa. The maintenance of an active zoonotic reservoir, reflected in the sustained circulation of the HPAI H5N1 virus in birds represents a formidable and persistent threat to public health, with significant regional spillover clusters. While human-to-human transmission remains limited overall, the occasional upward fluctuations increase in R₀ suggests the existence of localised episodes of amplified transmission risk at the human-animal interface.

The spatial heterogeneity, contrasting with moderate temporal variability, underscores that geographical context is a more potent predictor of risk than seasonal timing. This, combined with the uneven performance of surveillance systems, potentially compromises the timeliness of regional health responses. Overall, these results indicate that, despite the absence of pandemic spread, H5N1 poses a constant zoonotic pressure. This calls for operationalising geographically targeted One Health strategies. Continuous improvement of digital notification systems, standardised biosecurity, and locally adapted genomic research are essential to secure the African continent against future influenza-related threats.

## Data Availability

The datasets presented in this study can be found in online repositories. The names of the repository/repositories and accession number(s) can be found in the article/Supplementary Material.
